# HIV-1 Promotes Renal Tubular Epithelial Cell Protein Synthesis: Role of mTOR Pathway

**DOI:** 10.1371/journal.pone.0030071

**Published:** 2012-01-09

**Authors:** Shabina Rehman, Mohammad Husain, Anju Yadav, Balakuntalam S. Kasinath, Ashwani Malhotra, Pravin C. Singhal

**Affiliations:** 1 Department of Medicine, North Shore LIJ Health System, New York, New York, United States of America; 2 Department of Medicine, Texas Health Science Center, San Antonio, Texas, United States of America; South Texas Veterans Health Care System, United States of America

## Abstract

Tubular cell HIV-infection has been reported to manifest in the form of cellular hypertrophy and apoptosis. In the present study, we evaluated the role of mammalian target of rapamycin (mTOR) pathway in the HIV induction of tubular cell protein synthesis. Mouse proximal tubular epithelial cells (MPTECs) were transduced with either *gag*/*pol*-deleted NL4-3 (HIV/MPTEC) or empty vector (Vector/MPTEC). HIV/MPTEC showed enhanced DNA synthesis when compared with Vector/MPTECs by BRDU labeling studies. HIV/MPTECs also showed enhanced production of β-laminin and fibronection in addition to increased protein content per cell. In i*n vivo* studies, renal cortical sections from HIV transgenic mice and HIVAN patients showed enhanced tubular cell phosphorylation of mTOR. Analysis of mTOR revealed increased expression of phospho (p)-mTOR in HIV/MPTECs when compared to vector/MPTECs. Further downstream analysis of mTOR pathway revealed enhanced phosphorylation of p70S6 kinase and associated diminished phosphorylation of eEF2 (eukaryotic translation elongation factor 2) in HIV/MPTECs; moreover, HIV/MPTECs displayed enhanced phosphorylation of eIF4B (eukaryotic translation initiation factor 4B) and 4EBP-1 (eukaryotic 4E binding protein). To confirm our hypothesis, we evaluated the effect of rapamycin on HIV-induced tubular cell downstream signaling. Rapamycin not only attenuated phosphorylation of p70S6 kinase and associated down stream signaling in HIV/MPTECs but also inhibited HIV-1 induced tubular cell protein synthesis. These findings suggest that mTOR pathway is activated in HIV-induced enhanced tubular cell protein synthesis and contributes to tubular cell hypertrophy.

## Introduction

HIV-associated nephropathy (HIVAN) is characterized by tubular microcyst formation [1]. Tubular cells in these lesions display growth arrest, hypetrophy and apoptosis [2]. The role of mTOR in the development of cysts both in animal and human models of polycystic disease has been reported [3]–[6]. Recently, the role of mTOR has been suggested in the development of renal lesions in a mouse model of HIVAN [7]. In these studies, HIV transgenic mice (Tg26) displayed expression of phospho-mTOR by kidney cells. Renal tissues from Tg26 mice not only displayed enhanced phosphorylation of p70S6 kinase and eEF2K but also showed enhanced phosphorylation of 4E-BP-1 and eIF4B; these findings indicated the activation of mTOR pathway in kidney cells of HIV transgenic mice. However, whether mTOR activation contributes to the induction of tubular cell protein synthesis was not evaluated in those studies. Recent investigations have revealed that mTOR activation occurs in two distinct complexes: the mTOR complex1 (mTORC1) made up of mTOR, raptor, mSLT8, and, mTORC2 consisting of mTOR, rictor, diptor and mSLT8 [8]. Since mTORC1 plays an important role in the regulation of mRNA translation, a rate-limiting step in protein synthesis, we hypothesized that HIV-1 recruits mTOR for the induction of tubular cell protein synthesis.

mTORC1 is an important regulator of mRNA translation by two distinct but integrated pathways [8]-[10]. One track leads to the phosphorylation of ribosomal protein S6 by the ribosomal S6 kinase that stimulates the translation of mRNAs, which encode many components of the protein synthetic machinery- ribosomal proteins and translation initiation and elongation factors associated with regulating protein synthesis rates [9], [10]. The second track of the mTORC1-dependent pathway controls phosphorylation of 4EBP1 by releasing its inhibitory interaction with eIF4E, and thus allowing eIF4E to associate with eIF4G; the latter forms the active eIF4F complex that binds to the 5′ cap of the mRNA and facilitates the initiation phase of mRNA translation. eIF4E activity is important for the translation of transcripts of mRNAs encoding many proteins associated with growth and proliferation control such as cyclin D1 and c-myc [9], [10]. In mammalian cells, both 4EBP1/eIF4E and S6K are necessary for effective regulation of cell mass [9], [10].

In the recent years, activation of mTORC1 pathway has been shown in renal diseases in experimental animals and humans [11]–[15]. On that account, rapamycin, an inhibitor of mTOR pathway, has been used to attenuate s inflammation associated with allograft nephropathy in humans [15] and progression of renal lesions in membranous nephropathy, diabetic nephropathy, Thy 1.1 nephritis, and polycystic kidney disease in animal experimental models [11]–[14]. Recently, HIV transgenic mice-receiving rapamycin displayed attenuated renal lesions, proteinuria and uremia [7]. Moreover, in these studies renal tissues of HIVAN mice showed inhibition of the mTOR-associated downstream signaling. Thus, it appears that modulation of mTOR can be used as an effective therapeutic tool to provide protection against the progression of renal diseases in patients with HIVAN.

In the present study, we examined the effect of HIV-1 infection on the activation of mTORC1 pathway in mouse tubular cells and its outcome on tubular cell growth. In addition, we studied the effect of rapamycin, an inhibitor of mTORC1, on HIV-1-induced tubular cell mTORC1 pathway activation and associated effects on tubular cell protein synthesis and its protein content.

## Materials and Methods

### HIV transgenic mice (Tg26)

We used age and sex matched FVB/N (control) and Tg26 (on FVB/N background). Breeding pairs of FVBN were obtained from Jackson Laboratories (Bar Harbor, ME). Breeding pairs to develop Tg26 colonies were kindly gifted by Prof. Paul E. Klotman M.D., President and CEO, Baylor Medical College, Houston, TX). The Tg26 transgenic animal has the proviral transgene, pNL4-3: d1443, which encodes all the HIV-1 genes except *gag* and *pol;* therefore, the mice are noninfectious. Mice were housed in groups of 4 in a laminar-flow facility (Small Animal Facility, Long Island Jewish Medical Center, New Hyde Park, NY). We breed and maintain colonies of these animals in our animal facility.

### Human renal biopsy specimens

Archived renal biopsy specimens from patients with idiopathic focal glomerulosclerosis and HIVAN were obtained from the Pathology Department at our institution. Protocol for use of human tissue was approved by the Institutional Review Board of Feinstein Institute for Medical Research, Manhasset, NY.

### Proximal tubular cells

Mouse proximal tubular epithelial cells (MTC) were gift from Dr. G. Wolf (Department of Medicine, Hamburg, Germany). These cells are well characterized and expressed tubular cell markers [16].

### Production of Pseudotyped Retroviral Supernatant

Replication defective viral supernatants were prepared as published previously [17]. In brief, green fluorescence protein (GFP) reporter gene (from pEGFP-C1; Clontech, Palo Alto, CA) was substituted in place of *gag/pol* genes in HIV-1 proviral construct pNL4–3. This parental construct (pNL4-3: ΔG/P-GFP) was used to produce VSV.G pseudotyped viruses to provide pleiotropism and high-titer virus stocks. Infectious viral supernatants were produced by transient transfection of 293T cells using Effectene (Qiagen Inc.) according to the manufacturer's instructions. The HIV-1 *gag*/*pol* and VSV.G envelope genes were provided *in trans* using pCMV R8.91 and pMD.G plasmids, respectively (gifts of Dr. Didier Trono, Salk Institute, La Jolla, CA). As a negative control, virus was also produced from pHR-CMV-IRES2-GFP-ΔB, which contained HIV-1 LTRs and GFP empty expression vector. The viral stocks were titrated by infecting 293T cells with ten-fold serial dilution as reported previously [17]. The reciprocal of the lowest dilution showing the expression of GFP was defined as GFP-expressing units (GEU) per ml. Viral stocks ranging from 10^6^ to 10^8^ GEU/ml were obtained. Low-titer viral stocks were further concentrated by high speed centrifugation.

### Determination of cellular protein content

Equal numbers of MPTECs were transduced with empty vector or NL4-3 and then incubated in media for 72 hours. At the end of the incubation period, cells were harvested and counted by a hemocytometer. Protein was extracted, measured, and, protein content was calculated per cell.

### Determination of DNA synthesis

Equal numbers of EV/MPTECs and NL4-3/MPTECs were seeded in 96-well plates. Cells were labeled for 12 hours and analyzed as per manufacturer instruction (Calbiochem, La Jolla, CA).

### Immunohistochemical staining

The immunohistochemistry protocol used in the present study has been described previously [7]. The primary antibody- phospho-mTOR (1∶500, #2971, Cell Signaling Technology Inc., Danvers, MA)- was followed by the secondary antibody at 1∶250 dilution and then incubated in ABC reagent (Vector Laboratories, Burlingame, CA) for 30 minutes. Sections were washed thrice in PBS and placed in VECTOR Nova RED substrate kit SK-4800 (Vector Laboratories, Burlingame, CA) followed by counterstaining with methyl green.

### Western blotting studies

NL4-3/MPTECs and EV/MPTECs under control and experimental conditions were incubated in media for 72 hours. At the end of the incubation period, cells were harvested, lysed in RIPA buffer containing 50 mM Tris-Cl (pH 7.5), 150 mM NaCl, 1mM EDTA, 1% NP-40, 0.25% Deoxycholate, 0.1% SDS, 1X protease inhibitor cocktail (Calbiochem, Cocktail Set I), 1mM PMSF, and 0.2mM sodium orthovanadate. Protein concentration was measured by Bradford Assay (Bio-Rad , Hercules, CA). Total protein extracts (2 mg/ml) were separated on a 4-15% polyacrylamide (PAGE) gel and transferred onto a nitrocellulose membrane using Bio-Rad miniblot apparatus. The blots were blocked with 5% milk and 0.1% TWEEN 20 in 1X PBS for 60 min at room temperature and then hybridized with the antiphospho-mTOR (1∶500, Cell Signaling Technology Inc., Danvers, MA), antiphospho-p70S6K (1∶500, Abcam, Cambridge, MA), antiphospho-eEF2 (1∶500, rabbit polyclonal, Cell Signaling, antiphospho-4EBP1 (1∶500, rabbit polyclonal, Cell Signaling), phospho-eIF4B (1∶500, rabbit polyclonal, Cell Signaling), and antiphospho-UBF (1∶500, SantaCruz Biotechnology, Santa Cruz, CA) antibodies and subsequently treated with horseradish peroxidase labeled appropriate secondary antibodies. The blots were developed using a chemiluminescence detection kit (PIERCE, Rockford, IL) and exposed to X-ray film (Eastman Kodak Co., Rochester, NY). Equal protein loading and the protein transfer were confirmed by stripping and immunoblotting for actin protein using a polyclonal α-Actin antibody (I-19, SantaCruz) on the same Western blots.

### Statistical analysis

For comparison of mean values between two groups, the unpaired t test was used. To compare values between multiple groups, analysis of variance (ANOVA) was applied and a Bonferroni multiple range test was used to calculate a p-value. Statistical significance was defined as P<0.05.

## Results

### Tubular cells show enhanced tubular cell mTOR phosphorlyation in HIVAN Patients and HIVTg mice

**Figure 1:**
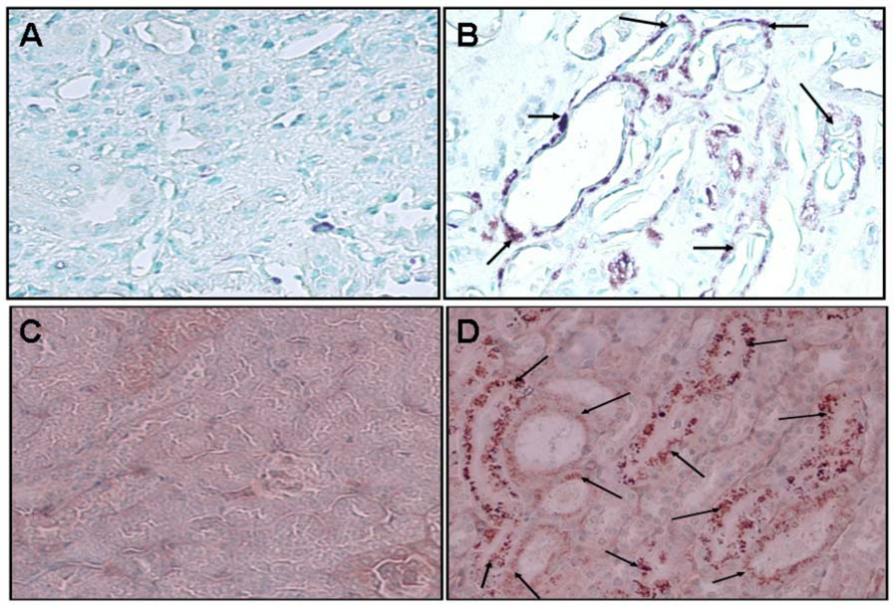
Tubular cells display enhanced phosphorlyation of mTOR in HIVAN. Renal biopsy specimens from patients with idiopathic focal and segmental glomerulosclerosis and HIVAN were immunolabeled for phospho-mTOR. Representative microphotograph from a patient with focal glomerulosclerosis (A) and a HIVAN patient (B) are shown. Renal tubular cells in dilated tubules displayed phosphorylation of mTOR (brown staining, indicated by arrows). Renal cortical sections of control (n = 3) and HIVAN (n = 3) mice were immunolabeled for phospho-mTOR and evaluated for tubular cell expression of phospho-mTOR. Representative microphotographs of a control (C) and HIVAN (D) mice are shown. Tubular cells in dilated tubules displayed enhanced phosphorylation of mTOR (brown staining, indicated by arrows).

Renal cortical sections of human biopsy specimens and HIVAN mice were immunolabeled for phospho-mTOR and evaluated for tubular cell expression of phospho-mTOR. As shown in Fig. 1, dilated tubules in both HIVAN patients and Tg26 mice displayed enhanced phospho-mTOR expression by tubular cells. These findings indicate the activation of mTOR pathway in diseased tubules in HIVAN patients and Tg26 mice.

### HIV promotes mTOR pathway activation in Tubular cells

**Figure 2:**
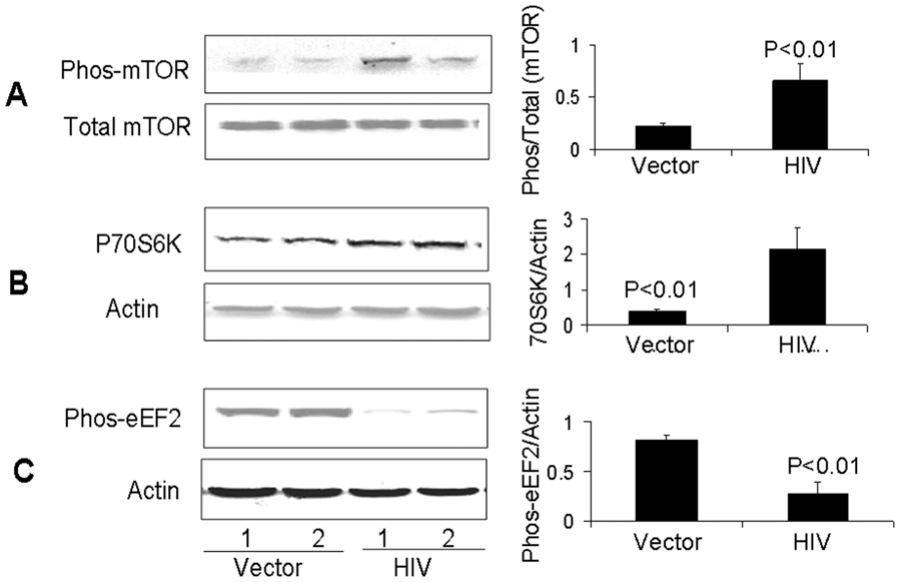
HIV induces mTOR phosphorylation and downstream signaling in Tubular cells. MPTECs were tranduced either with empty vector or NL4-3 and then incubated in media for 72 hours. Subsequently, proteins were extracted from vector/MPTECs and HIV/MPTECs, Western blots were prepared and probed for phospho-mTOR, p70S6K, and phospho-eEF2. Immunoblots were stripped and reprobed either for total mTOR or actin (n = 4). A. Representative gels (in duplicate) showing tubular cell expression of phospho-mTOR under control (vector) and HIV infection states (upper lane). The lower lane shows tubular cell expression of total mTOR under similar conditions. A bar diagram showing cumulative data of 4 sets of experiments is shown in the right panel. B. Representative gels (in duplicate) showing tubular cell expression of phospho-p70S6K under control (vector) and HIV infection states (upper lane). The lower lane shows tubular cell expression of actin under similar conditions. A bar diagram showing cumulative data of 4 sets of experiments is shown in the right panel. C. Representative gels (in duplicate) showing tubular cell expression of phospho-eEF2 under control (vector) and HIV infection states (upper lane). The lower lane shows tubular cell expression of actin under similar conditions. A bar diagram showing cumulative data of 4 sets of experiments is shown in the right panel.

**Figure 3:**
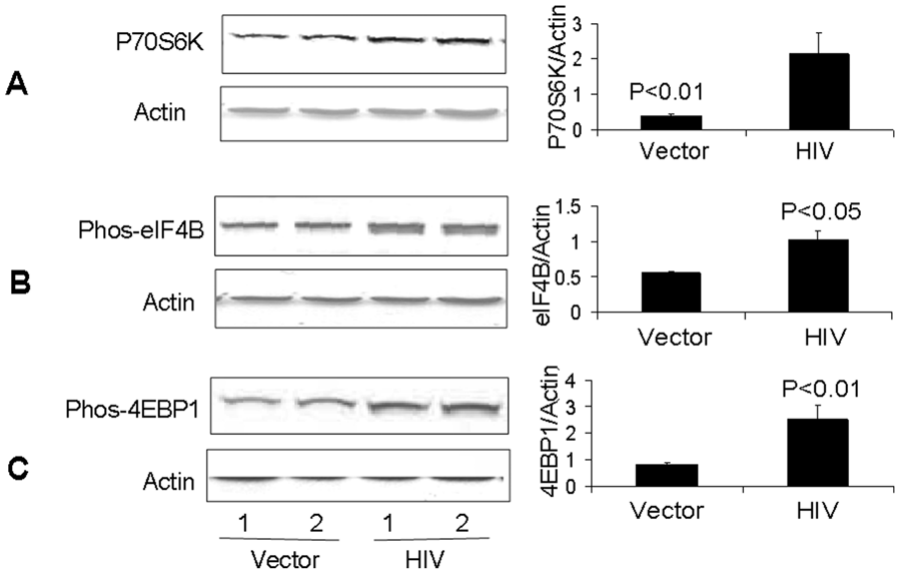
HIV induces phosphorylation p70S6K and downstream signaling in Tubular cells. MPTECs were tranduced either with empty vector or NL4-3 and then incubated in media for 72 hours. Subsequently, proteins were extracted from vector/MPTECs and HIV/MPTECs, Western blots were prepared and probed for phospho-70S6K, phospho-eIF4B, and phospho-4EBP1. Immunoblots were stripped and reprobed for actin (n = 4). A. Representative gels (in duplicate) showing tubular cell expression of phospho-70S6K under control (vector) and HIV infection states (upper lane). The lower lane shows tubular cell expression of actin under similar conditions. A bar diagram shows cumulative data of four sets of experiments in the right panel. B. Representative gels (in duplicate) showing tubular cell expression of phospho-eIF4B under control (vector) and HIV infection states (upper lane). The lower lane shows tubular cell expression of actin under similar conditions. A bar diagram showing cumulative data of four sets of experiments is in the right panel. C. Representative gels (in duplicate) showing tubular cell expression of phospho-EBP1 under control (vector) and HIV infection states (upper lane). The lower lane shows tubular cell expression of actin under similar conditions. A bar diagram showing cumulative data of 4 sets of experiments is shown in the right panel.

To determine the activation of mTOR pathway, lysates from vector/MPTECs and HIV/MPTECs were prepared for Western blotting and probed for the expression of proteins molecules involved in the downstream signaling of the mTORC1 pathway (n = 4). Representative gels (in duplicate) are shown in Fig. 2. Immunoblots of HIV/MPTECs showed increase in phos (Ser^2448^) of mTOR and in phos (Thr^389^) of p70S6 kinase, demonstrating the activation of mTORC1 in HIV/MPTECs. Moreover, reduction in eEF2 phos (Thr^56^) is indicative of increase in p70S6 kinase activation and stimulation of elongation phase of mRNA translation in proximal tubular epithelial cells [18]. Cumulative data (n = 4) are represented by bar graphs. Similarly, enhanced phosphorylation of 4EBP1 and eIF4B in HIV/MPTECs (Fig. 3) indicates activation of the initiation phase of mRNA translation and further confirms the activation of the mTORC1 pathway in HIV/MPTECs. Cumulative data (n = 4) are represented by bar graphs. Thus, HIV/MPTECs display evidence of mTOR activation and the stimulation of both initiation and elongation phases of mRNA translation.

### HIV/MPTECs display enhanced phosphorylation of UBF (upstream binding factor)

**Figure 4:**
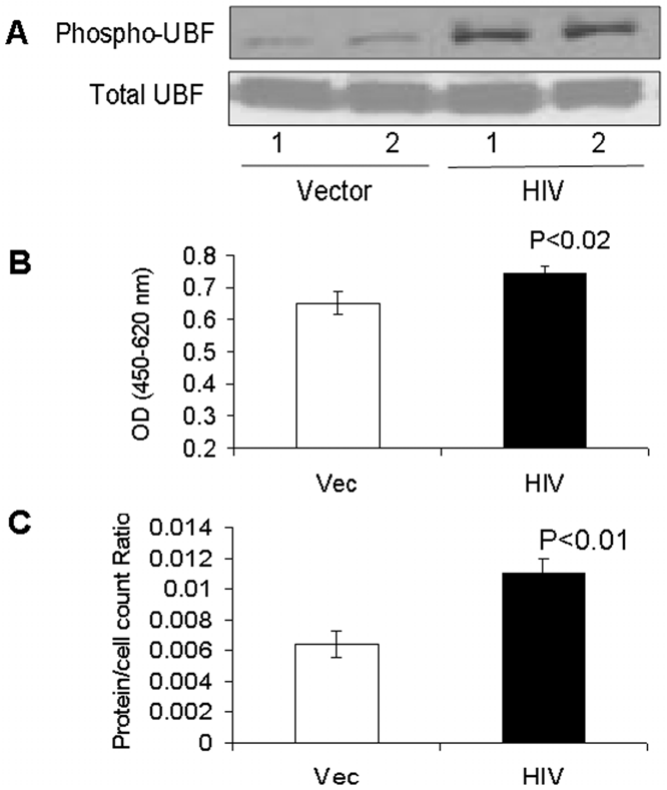
HIV enhances tubular cell UBF phosphorylation, DNA synthesis and intracellular protein content. A. Vector/MPTECs and HIV/MPTECs were incubated in media for 72 hours. Subsequently, proteins were extracted and probed for phospho-UBF and total UBF. Representative gels (in duplicate) are shown displaying tubular cell expression of phospho-UBF and toral UBF. The upper lane shows the effect of HIV on tubular cell expression of phospho-UBF. The lower lane shows lane total tubular cell UBF expression under similar conditions. B. Vector/MPTECs (control) and HIV/MPTECs were incubated 96 well plates and pulsed with BRDU and incubated for 72 hours. BRDU incorporation in MPTECs was assayed by ELISA. Cumulative data of three sets of experiments is show in the form a bar diagrams C. Vector/MPTECs and HIV/MPTECs were growth arrested and then incubated in media containing 1% serum for 72 hours. At the end of the incubation period, cells were harvested, total number of cells were counted and proteins were extracted. Protein content per cell was calculated. Cumulative data are displayed as bar graphs.

Regulation of mRNA translation can occur at the level of efficiency and/or capacity. The latter depends on the generation of more ribosomes, which would ultimately permit the cell to rapidly ramp up protein synthesis [19]. Since stimulation of ribosomal DNA (rDNA) transcription by mTOR is mediated in part through the phosphorylation of the carboxy-terminal activation domain of the rDNA transcription factor, upstream binding factor (UBF) [20], we also evaluated the effect of HIV infection on phospho-UBF expression by tubular cells. Lysates were prepared from vector/MPTECs and HIV/MPTECs and proteins were probed for phospho-UBF and total UBF. As shown in Fig. 4A, HIV/MPTECs displayed enhanced expression of phospho-UBF. These findings indicate that HIV may be stimulating rDNA transcription and ribosomal biogenesis in tubular cells through the activation of UBF.

### HIV enhances tubular cell DNA synthesis and increases intracellular protein content

Since mTORC1 pathway enhances protein synthesis, we determined whether the activation of HIV-induced mTOR pathway is associated with enhanced protein synthesis in MPTECs. Control and HIV/MPTECs were pulsed with BRDU and BRDU incorporation was assayed by ELISA. As shown in Fig. 4B, HIV/MPTECs displayed increased (P<0.02) BRDU labeling (DNA synthesis). In addition, vector/MPTECs and HIV/MPTECs were growth arrested and then stimulated to grow for 48 hours. Intracellular protein content was calculated by measuring total number of cells and total amount of protein in vector/MPTECs and HIV/MPTECs. As shown in Fig. 4C, HIV/MPTECs displayed increased (P<0.01) protein content per cell (n = 3). These data show that HIV promoted hypertrophy of tubular cells.

### Rapamycin inhibits HIV-induced tubular cell mTOR pathway signaling

**Figure 5:**
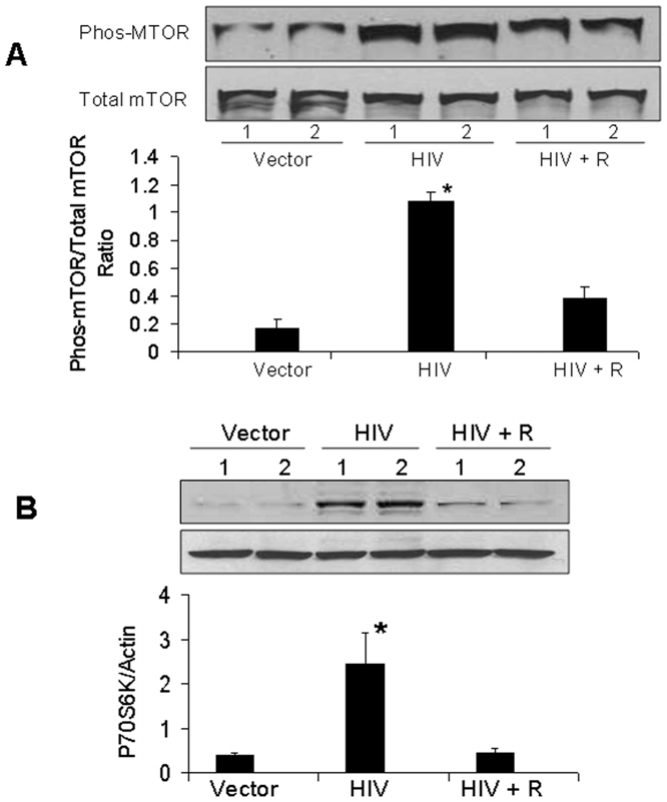
Rapamycin inhibits HIV-induced tubular cell phosphorylation of mTOR and 70S6K. A. MPTECs were transduced either empty vector (Vector), NL4-3 (HIV) and incubated in media containing either buffer or rapamycin (100 nM) for 72 hours. Subsequently, proteins were extracted, Western blots were prepared and probed for phospho-mTOR. The blots were stripped and reprobed for total mTOR. Representative gels (in duplicate) showing tubular cell phospho-mTOR in control (vector), HIV infected (HIV) and rapamycin-treated/HIV-infected (HIV + R) cells are shown (upper lane). The lower lane shows tubular cell expression of mTOR under similar conditions. Cumulative data of four sets of experiments in the form of a bar diagram are displayed in the lower panel. P<0.01 compared to vector and HIV + R. B. Proteins from the MPTECs treated under similar condition were probed for phospho-70S6K. The blots were stripped and reprobed for actin. Representative gels (in duplicate) showing tubular cell phospho-70S6K in control (vector), HIV infected (HIV) and rapamycin-treated/HIV-infected (HIV + R) cells are shown (upper lane). The lower lane shows tubular cell expression of actin under similar conditions. Cumulative data of four sets of experiments in the form of a bar diagram are displayed in the lower panel. *P<0.01 compared to vector and HIV + R.

To determine the effect of rapamycin on HIV-induced tubular cell mTOR phosphorylation, proteins were extracted from vector/MPTECs and HIV/MPTECs, and HIV + R/MPTECs and protein lysates probed for the expression of phospho-mTOR and total mTOR. Representative gels (in duplicate) are shown in Fig. 5A. Cumulative data (n = 4) are shown in the form of graphical representation. HIV/MPTECs displayed enhanced (P<0.01) mTOR phosphorylation; however, rapamycin inhibited (P<0.01) this effect of HIV on tubular cells.

Immunoblots prepared under above mentioned conditions were probed for the expression of phospho-p70S6K and actin. Representative gels (in duplicate) are shown in Fig. 5B. Cumulative data (n = 4) are represented by a bar diagram. HIV/MPTECs displayed enhanced (P<0.01) expression of phos-p70S6K that was completely inhibited by rapamycin (P<0.01).

**Figure 6:**
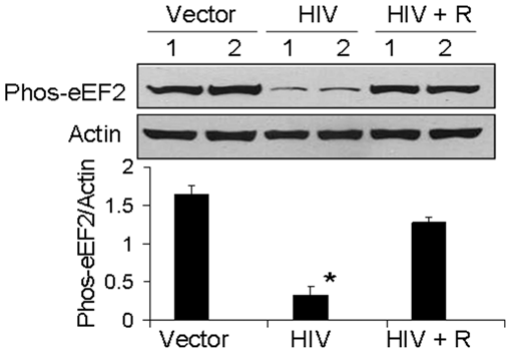
Rapamycin inhibits HIV-induced tubular cell phosphorylation of eEF2. MPTECs were transduced either empty vector (Vector), NL4-3 (HIV) and incubated in media containing either buffer or rapamycin (100 nM) for 72 hours. Subsequently, proteins were extracted, Western blots were prepared and probed for phospho-eEF2. Immunoblots were stripped and reprobed for actin mTOR. Representative gels (in duplicate) showing tubular cell phospho-eEF2 in control (vector), HIV infected (HIV) and rapamycin-treated/HIV-infected (HIV + R) cells are shown (upper lane). The lower lane shows tubular cell expression of actin under similar conditions. Cumulative data of four sets of experiments in the form of a bar diagram are displayed in the lower panel. *P<0.01 compared to vector and HIV + R.

Similarly, immunoblots were probed for the expression of phospho-eEF2 and actin. Representative gels (in duplicate) are shown in Fig. 6. Cumulative data (n = 4) are shown as bar graphs. HIV/MPTECs displayed diminished (P<0.01) expression of phospho-eEF2 that was restored by rapamycin (P<0.01).

**Figure 7:**
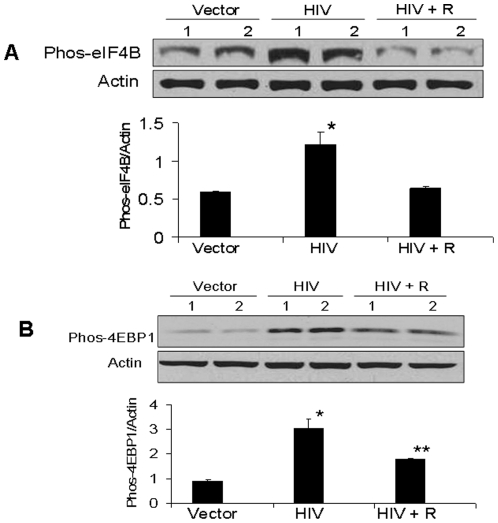
Rapamycin inhibits HIV-induced tubular cell phosphorylation of eIF4B and EBP1. A. MPTECs were transduced either empty vector (Vector), NL4-3 (HIV) and incubated in media containing either buffer or rapamycin (100 nM) for 72 hours. Subsequently, proteins were extracted, Western blots were prepared and probed for phospho-eIF4B. Immunoblots were stripped and reprobed for actin. Representative gels (in duplicate) showing tubular cell phospho-eIF4B in control (vector), HIV infected (HIV) and rapamycin-treated/HIV-infected (HIV + R) cells are shown (upper lane). The lower lane shows tubular cell expression of actin under similar conditions. Cumulative data of four sets of experiments in the form of a bar diagram are displayed in the lower panel. *P<0.01 compared to vector and HIV + R. B. Proteins from the MPTECs treated under similar conditions were probed for phospho-4EBP1. The blots were stripped and reprobed for actin. Representative gels (in duplicate) showing tubular cell phospho4EBP1 in control (vector), HIV infected (HIV) and rapamycin-treated/HIV-infected (HIV + R) cells are shown (upper lane). The lower lane shows tubular cell expression of actin under similar conditions. Cumulative data of four sets of experiments in the form of a bar diagram are displayed in the lower panel. *P<0.001 compared to vector. **P<0.01 compared to HIV + R.

Immunoblots were also probed for the expression of phospho-eIF4B and actin. eIF4B is believed to assist the helicase action of eIF4A during the initiation phase of translation; it is activated by phosphorylation by p70S6 kinase [21], which, in turn, is dependent on mTORC1. Representative gels (in duplicate) are shown in Fig. 7A. Cumulative data (n = 4) in the form of a bar diagram is also shown. HIV/MPTECs displayed enhanced (P<0.01) expression of phos-eIF4B; again, this was neutralized by rapamycin (P<0.01). Similar effect of rapamycin was seen in HIV-induced increase in 4EBP1 phosphorylation (Fig. 7B, p<0.01).

### Rapamycin inhibits HIV-induced tubular cell β-laminin and fibronectin synthesis

**Figure 8:**
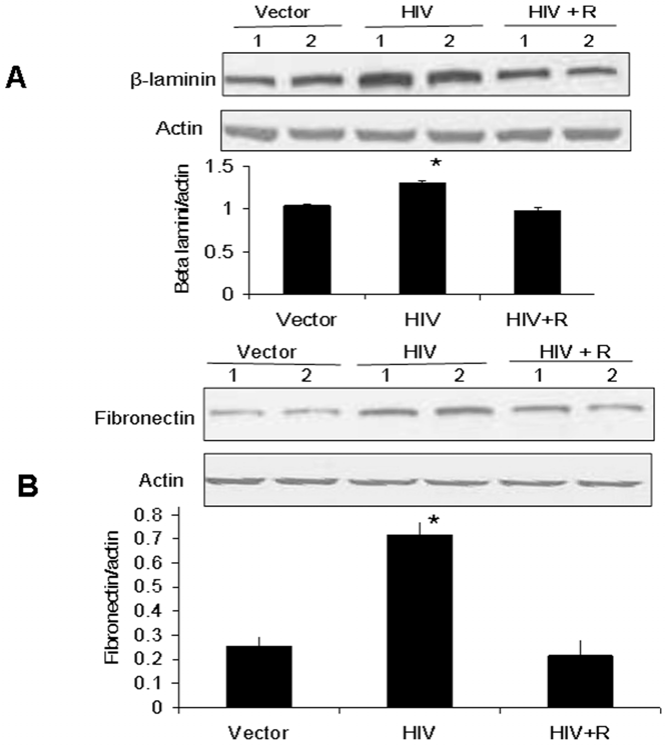
Rapamycin inhibits HIV-induced tubular cell β-laminin and fibronectin synthesis. A. MPTECs were transduced either empty vector (Vector), NL4-3 (HIV) and incubated in media containing either buffer or rapamycin (100 nM) for 48 hours. Subsequently, proteins were extracted, Western blots were probed for β-laminin1. Immunoblots were stripped and reprobed for actin. Representative gels (in duplicate) showing tubular cell β-laminin1 in control (vector), HIV infected (HIV) and rapamycin-treated/HIV-infected (HIV + R) cells are shown (upper lane). The lower lane shows tubular cell expression of actin under similar conditions. Cumulative data of four sets of experiments in the form of a bar diagram are displayed in the lower panel. *P<0.01 compared to vector and HIV + R. B. Proteins from the MPTECs treated under similar conditions were probed for fibronectin. The blots were stripped and reprobed for actin. Representative gels (in duplicate) showing tubular cell fibronectin in control (vector), HIV infected (HIV) and rapamycin-treated/HIV-infected (HIV + R) cells are shown (upper lane). The lower lane shows tubular cell expression of actin under similar conditions. Cumulative data of four sets of experiments in the form of a bar diagram are displayed in the lower panel. *P<0.001 compared to vector and HIV + R.

Activation of mTOR pathway enhances the synthesis of several matrix proteins amongst them β-laminin1 and fibronectin are main targets. To evaluate the effect of rapamycin on HIV-induced tubular cell β-laminin synthesis, proteins were extracted from vector/MPTECs, HIV/MPTECs, and HIV + R/MPTECs. Western blots were probed for β-laminin1 and actin. Representative gels (in duplicate) are shown in Fig. 8A. Cumulative data (n = 4) are shown as bar diagrams. HIV/MPTECs displayed enhanced (P<0.01) expression of β-laminin1; rapamycin inhibited (P<0.01) the HIV-induced increment.

Similarly, analysis of fibronectin through Western blots revealed enhanced fibronectin synthesis in tubular cells exposed to HIV that was completely abolished by rapamycin (Fig. 8B, p<0.01).

### Rapamycin inhibits HIV-induced tubular cell protein synthesis

**Figure 9:**
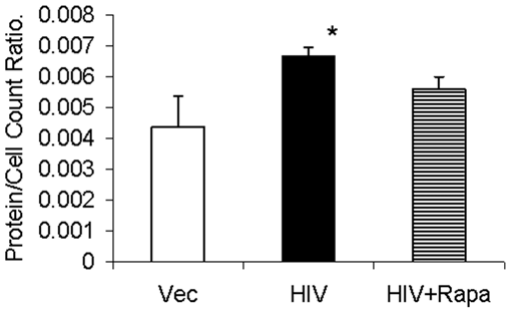
Effect of rapamycin on HIV-induced tubular cell protein synthesis. Growth arrested vector/MPTECs, and HIV/MPTECs with or without rapamycin (100 nM) were incubated in media for 48 hours. Subsequently, number of cells were counted and total proteins were measured quantitatively. Protein content per cell was calculated (n = 3). P<0.05 compared to vector and HIV + R.

To determine the effect of mTOR pathway inhibition on HIV-induced tubular cell protein synthesis, vector/MPTECs, HIV/MPTECs, and HIV + R/MPTECs were growth arrested and then allowed to grow for 48 hours. Intracellular protein content per cell was calculated by measuring total number of cells and total amount of protein. As shown in Fig. 9, HIV/MPTECs displayed increased (P<0.05) protein content per cell. However, rapamycin inhibited (P<0.05) this effect of HIV on tubular cell protein synthesis.

## Discussion

In the present study, HIV/MPTECs showed occurrence of enhanced DNA synthesis in the form of increased BRDU labeling. In addition, HIV/MPTECs showed enhanced expression of β-laminin, fibronectin, and increased amount of protein content per cell. Immunoblot data of HIV/MPTECs showed enhanced in phos (Ser^2448^) of mTOR and in phos (Thr^389^) of p70S6 kinase; these findings indicated the activation of mTORC1 pathway in HIV-1-infected tubular cells. Similarly, a reduction in eEF2 phos on Thr^56^ also indicated the activation of p70S6 kinase and stimulation of elongation phase of mRNA translation in HIV-1-infected tubular cells. Enhanced phosphorylation of 4EBP1 and eIF4B in HIV/MPTECs strongly suggests stimulation of the initiation of mRNA translation phase. These findings indicated the activation of mTORC1 pathway in HIV-infected tubular cells. However, immunoblot analyses of data from rapamycin-treated HIV/MPTEC demonstrated attenuated phos (Thr^389^) of p70S6K thus, suggesting inhibition of mTORC1. Further, protein expression of rapamycin treated HIV/MPTEC showed an increase in eEF2 phos of Thr^56^ (when compared to HIV/MPTECs), which is indicative of inhibition of p70S6 kinase and associated inhibition of the elongation phase of mRNA translation. Rapamycin treated HIV/MPTECs not only attenuated expression of β-laminin and fibronectin but also displayed attenuated protein content when compared to HIV/MPTECs.

The activation of the mTOR pathway has been reported to contribute to the pathogenesis of cystic kidney diseases [3], [4]. Induction of phospho-mTOR and p70S6K has been demonstrated in cyst-lining epithelial cells in cysts both from mouse and human kidneys [4]. Han:SPRD/PKD rat kidneys showed enhanced expression of p70S6K (Thr^389^) and total S6K and thus indicating the activation of the mTOR pathway [3]; moreover, rapamycin inhibited the activation of the mTOR pathway in kidneys of Han:SPRD rats. In the present study, we observed enhanced tubular cell phosphorylation of mTOR in dilated tubules of HIVAN patients as well HIVAN mice. These findings are consistent with the observations of other investigators indicating the activation of the mTOR pathway in epithelial lining of cystic tubules.

The mTOR pathway responds to growth factors and mitogens and stimulates cap-dependent translation, whereas amino-acid deprivation and hypoxic stress down regulate this pathway and thus leading to a reduction in global protein synthesis [22]–[26]. Interestingly, in the present study, HIV hijacked kidney cell genome to activate mTOR pathway to enhance protein translation needed for viral replication. The mTOR exists in two complexes: mTORC1, which is rapamycin-sensitive and promotes downstream signaling through the phosphorylation of p70S6K and eukaryotic initiation factor 4E binding proteins (4EBPs), whereas, mTORC2 is rapamycin-insensitive and phosphorylates AKT [18]. Although both mTOR complexes are stimulated by mitogens, but only mTORC1 is under the control of nutrient and energy inputs. Down regulation of mTOR pathway is associated with resistance to oxidative, osmotic, hypoxic and apoptotic stresses [22]–[26]. On the other hand, over-activation of the mTOR pathway leads to higher than normal protein synthesis in several disease states. Increase in phosphorylation of Thr389 on p70S6 kinase and Thr37/46 on 4E-BP1 are indices of activation of mTORC1. This was further supported by the ability of rapamycin to inhibit these events in addition to general protein synthesis induced by HIV.

Loss of critical neprhon mass was demonstrated to be associated with progressive renal failure both in animal and human disease models of kidney diseases (27]. Initially, the loss of nephrons was associated with compensatory renal hypertrophy [27]–[29]. The mTOR was implicated as the major pathway which contributed to renal hypertrophy [30]. Activation of the mTOR pathway was displayed in the form of enhanced renal tissue phosphorylation of p70S6K and 4EBP1 in the remaining kidney in a uni-nephrectomy model [30]; conversely, inhibition of the mTOR pathway by rapamycin in this model was associated with attenuation of renal hypertrophy [30]. Moreover, in mice with genetically engineered deletion of p70S6 kinase, renal hypertrophy was not seen either with diabetes or following uninephrectomy [31]. Interestingly, rapamycin inhibited tubular dilatation and interstitial volume in a unilateral ureteral obstruction (UUO) mouse model [32]. In the latter model, rapamycin not only diminished the infiltration of inflammatory cells but also attenuated renal tissue transforming growth factor-β1 expression. The authors suggested the role the mTORC1 pathway activation in the progression of tubulo-interstitial injury and fibrosis in obstructive uropathy [32]. Similarly, rapamycin not only inhibited mTOR-induced downstream signaling but also attenuated the progression of tubular lesions in a mouse model of HIVAN [7]. In the present study, rapamycin attenuated the HIV-1- induced tubular cell activation of the mTORC1 pathway as well as tubular cell protein synthesis; these findings provide evidence that mTORC1 contributes to tubular cell hypertrophy during HIV infection.

In many disease conditions despite mTORC1 activation, the outcome in terms of enhanced protein synthesis may be completely different. For example, the activation of the mTOR pathway has been shown to occur in both human and mouse lupus nephritis [33], and anti-Thy1.1-induced chronic glomerulosclerosis in the rat [34], [35], but these nephritic lesions were associated with a reduction in protein synthesis due to the activation of protein kinase R (PKR)-like ER kinases (PERK) and phosphorylation of eIF2, as a compensatory mechanism [36]–[38]. Thus, it appears that protein expression is the net outcome of competing signaling pathways working for enhancement vs. reduction in protein translation. Therefore, like any other growth pathway, the effect of the activation of the mTOR pathway on the outcome- protein synthesis- will be dependent on the net balance between the activation of competing pathways for upregulation and downregulation of protein translation.

The mTORC1 activation may not yield net outcome in the form of enhanced protein synthesis and cellular hypertrophy at all stages of disease, depending on the stimulatory and inhibitory factors mentioned above. Diabetic nephropathy is characterized by renal hypertrophy in general, and glomerular hypertrophy in particular [39]. However, renal hypertrophy occurred during the first month following diabetes induction in streptozotocin-treated rats because of enhanced cap-dependent protein synthesis through the activation of mTORC1 [28], [29]; however, in the latter time periods, cap-dependent protein synthesis was down regulated in response to an ongoing oxidative [40] and ER stresses [41], [42]; these stresses inhibited protein synthesis, and thus tilted the balance towards negative protein balance.

Recently, the role of mTOR pathway has been demonstrated in the development of HIVAN proliferative phenotype in cystic tubules of HIVAN mice [7]; in addition to tubular cell proliferation, occurrence of apoptosis was also displayed in microcysts. Thus, it appears that in HIVAN too, mitogenic vs. apoptotic pathways dominate at different time points. However, the time course effect of mTOR pathway on the expression of different protein expression profile was not evaluated in those studies. Therefore, it will be interesting to evaluate the time course effect of HIV-1 infection on the expression of renal tissue protein profile in HIVAN in future studies.

Besides inhibition of mTOR pathway, rapamycin has also been reported to display anti-HIV properties (43–47]. Roy et al demonstrated that rapamycin inhibited LTR-mediated transcription of HIV [43]. Recently, Heredia et al demonstrated that rapamycin down regulated CCR5 and caused accumulation of anti-HIV β-chemokines [44]. Moreover, *in vitro* studies, rapamycin enhanced the anti-HIV activity of HIV-entry inhibitors including vivcriviroc, aplaviroc and enfuvirtide [44], [45]. In addition, rapamycin attenuated HIV infection in human peripheral blood leukocyte reconstituted SCID mice [46]. Furthermore, in a recent prospective trial, rapamycin (as a monotherapy) showed significantly better control of HIV and hepatitis C virus replication in HIV patients with liver transplant [47]. Thus, it appears that rapamycin not only attenuates HIV replication but also inhibits its entry target cells expressing CCR5.

**Figure 10:**
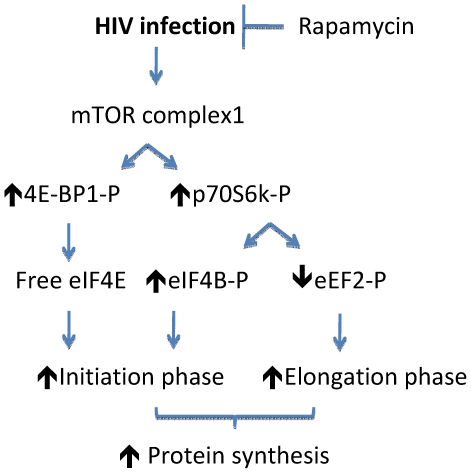
Proposed mechanism of rapamycin-induced inhibition of HIV-induced protein synthesis.

A schema proposing potential mechanism by which mTORC1 promotes protein synthesis in tubular epithelial cells in HIVAN is shown in Fig. 10. The HIV-induced mTORC1 activation proceeds with the rapamycin-sensitive phosphorylation of 4EBP1 which by freeing eIF4E promotes the initiation phase of mRNA translation. Activation of mTORC1 leads to the phosphorylation of p70S6 kinase, which phosphorylates eIF4B on Ser^422^ [21]. During the initiation phase of translation, eIF4A, a DEAD box protein, functions as a helicase resolving the complexities in 5″UTR which facilitates the scanning of the pre-initiation complex for the AUG codon; eIF4B assists in this activity [9]. Additionally, p70S6 kinase inhibits the activity of eEF2 kinase, a calcium calmodulin–dependent kinase, by phosphorylating it on Thr^366^ [48]; reduced activity of eEF2 kinase would contribute to increase in the level of eEF2 dephosphorylated on Thr^56^; this results in activation of eEF2 [49]. These events stimulate the elongation phase of mRNA translation. Thus, the activation of mTORC1 pathway contributes to the translation of mRNA by stimulating the initiation as well as the elongation phases. By targeting mTORC1, rapamycin inhibits the two critical pathways in mRNA translation that together promote protein synthesis.

We conclude that HIV-1 infection of tubular cells induces activation of the mTORC1 pathway leading to stimulation protein synthesis and thus contributing to tubular cell hypertrophy. Rapamycin inhibits tubular cell protein synthesis by inhibting the activation of the mTORC1. The present study provides insight into the molecular mechanisms involved in tubular cell hypertrophy in HIVAN.
